# Ocular inflammatory events and COVID-19 vaccination: correspondence

**DOI:** 10.1186/s12348-023-00371-0

**Published:** 2023-11-08

**Authors:** Pathum Sookaromdee, Viroj Wiwanitkit

**Affiliations:** 1 Bangkok, Thailand; 2 Dr DY Patil Vidhyapeeth, Pune, India

Dear Editor, we read the article entitle “Ocular inflammatory events following COVID-19 vaccination: a multinational case series [1]” with a great interest. Testi et al. concluded that “*Ocular inflammatory events may occur after COVID-19 vaccination. The findings are based on a temporal association that does not prove causality. Even in the possibility of a causal association, most of the events were mild and had a good visual outcome* [1].” We agree that there is a risk of ocular side effects following COVID-19 vaccination. There is lno doubt that some COVID-19 patients may experience a typical pathogenic immunological response, which may be linked to ocular inflammation. Despite this, most published cases of post-vaccination ocular inflammation lack information on pre-vaccination immunological and ocular state. Other underlying causes that could cause ocular inflammatory condition after immunization are also plausible. After vaccination, change of blood viscosity occur and might be related with clinical problem [2]. The ocular problem related to COVID-19 vaccine induced is also mentioned and it might explain the mild nature of ocular problem [3]. Finally, there is also a possibility of concurrent medical disorder in a COVID-19 vaccine recipient. Dengue is a good example [4]. Dengue is also a possible cause of ocular inflammation [5] and might coexist in a vaccine recipient.

## Data Availability

There is no associated data for this correspondence.
